# Brexit and bots: characterizing the behaviour of automated accounts on Twitter during the UK election

**DOI:** 10.1140/epjds/s13688-022-00330-0

**Published:** 2022-03-22

**Authors:** Matteo Bruno, Renaud Lambiotte, Fabio Saracco

**Affiliations:** 1 IMT School for Advanced Studies, P.zza S. Francesco 19, 55100 Lucca, Italy; 2 Mathematical Institute, University of Oxford, Woodstock Road, OX2 6GG Oxford, UK; 3 Institute for Applied Mathematics, National Research Council, Via dei Taurini 19, 00185 Rome, Italy

**Keywords:** Social networks, Bots, Misinformation

## Abstract

Online Social Networks (OSNs) offer new means for political communications that have quickly begun to play crucial roles in political campaigns, due to their pervasiveness and communication speed. However, the OSN environment is quite slippery and hides potential risks: many studies presented evidence about the presence of d/misinformation campaigns and malicious activities by genuine or automated users, putting at severe risk the efficiency of online and offline political campaigns. This phenomenon is particularly evident during crucial political events, as political elections. In the present paper, we provide a comprehensive description of the networks of interactions among users and bots during the UK elections of 2019. In particular, we focus on the polarised discussion about Brexit on Twitter, analysing a data set made of more than 10 millions tweets posted for over a month. We found that the presence of automated accounts infected the debate particularly in the days before the UK national elections, in which we find a steep increase of bots in the discussion; in the days after the election day, their incidence returned to values similar to the ones observed few weeks before the elections. On the other hand, we found that the number of suspended users (i.e. accounts that were removed by the platform for some violation of the Twitter policy) remained constant until the election day, after which it reached significantly higher values. Remarkably, after the TV debate between Boris Johnson and Jeremy Corbyn, we observed the injection of a large number of novel bots whose behaviour is markedly different from that of pre-existing ones. Finally, we explored the bots’ political orientation, finding that their activity is spread across the whole political spectrum, although in different proportions, and we studied the different usage of hashtags and URLs by automated accounts and suspended users, targeting the formation of common narratives in different sides of the debate.

## Introduction

Even if the extent of the impact on election results and on public opinion about pivotal political topics is debated [1–3], new media undeniably represent an extensively used novel channel that has contributed to reshape the political communication strategies. Therefore, due to their social relevance and the technical interest, researchers from different disciplines have accordingly focused on the description and analysis of social media structure and dynamics, shedding light on non trivial behaviours occurring in debates in online social networks [4–10]. For instance, the topic of echo chambers, i.e. the creation of polarized groups of users based on the access to similar news sources [11], has received great attention. Even if similar phenomena were already observed in offline contexts before the advent of OSNs [12], echo-chambers’ effects seem to be amplified in social networks [7, 11]. In particular, many contributions showed how the formation of echo chambers depends extremely on the kind of platform analysed, showing that some of them -for instance, Twitter or Facebook- are more prone to segregate accounts in groups with similar opinions [13–15]. Nevertheless, there is no complete agreement on the existence or even on the role of echo-chambers in the online debate [1, 16–18].

Beside the different mechanism of opinion dynamics due to the different structures of social networks platforms and the various possibilities for users to interact with each other, it is of crucial importance to investigate artificial manipulations of the behaviour of users. For instance, in recent years a great part of the research literature on social networks has been dedicated to the analysis of the behaviour of social bots (or simply *bots*), i.e. automated social accounts controlled by pieces of software. In general, they can be used to automatically share the latest news or provide information in case of emergencies. Nonetheless, in many occasions malicious activities by automated accounts were observed, with bots often hiding and pretending to be human [5, 8]. Among other usages, it was showed that their employment may foster, precisely, the formation of echo chambers [19] or increase the visibility and credibility of users [6, 8, 20, 21]. The injection of coordinated accounts is particularly common during elections and key events in several countries [22–24]. Detecting the bots and analysing the behaviour of malicious accounts is not, however, an easy task. For now more than a decade, scientists have worked on the detection of automated accounts, using a variety of approaches involving machine learning to analyse linguistic and behavioural features [8, 25, 26] and the analysis of social interactions [27, 28].

In this paper, we focus on the discussion about Brexit on Twitter before and during the UK elections of 2019. The presence and influence of automated accounts in the Twitter Brexit discussion was already noted during the 2016 referendum: in their work, Bastos and Mercea [29] found a botnet of more than 10k bots, propagating fake and hyperpartisan news. They found that the bots are specialising in different activities, with some of them retweeting active users and some other generating cascades of information from other bots’ tweets. Similarly, the work of Howard and Kollanyi [30] showed that the bots were mainly focused on retweeting and that their activity was very high compared to genuine users. Furthermore, they found that the bots’ activity was more in support of Brexit than against it.

Here, we focus on the UK elections in 2019, as they played a pivotal role for the confirmation of Brexit^1^ and they polarised the public opinion about the future relations of the UK with the European Union. To investigate this matter, we have analysed a dataset that includes more than 10 million Brexit-related tweets and 1 million users, over a period of more than one month centered on the day of the general election, held on the 12th of December 2019.

We summarize our Research Objectives (ROs) as follows.

- *RO1*: quantify the presence of bots talking about Brexit during the UK election process.
- *RO2*: understand the bots’ strategies in terms of their positions in the networks of retweets.
- *RO3*: uncover the main Brexit subtopics on which the bots focus most.
- *RO4*: unveil the similarities and differences between suspended users and accounts labeled as bots.

For RO1, we check for the presence and the activity patterns of the bots for our time window centered on the UK election day. Dealing with RO2, by building networks of retweets we are able to see if the bots spread equally through the political groups and if they are positioned in the core or periphery of the network. For RO3, we analyse the hashtags and URLs used mostly by the bots. We also build networks of bots by linking those that show similar patterns of hashtags/URLs usage to find coordinated activity by automated accounts. To tackle RO4, we analyse the categories of bots and suspended users separately for the previous ROs.

## Methods

### Data collection

Twitter is commonly adopted in the UK, where it is the second online social network platform after Facebook, according to Statista:^2^ Twitter accounts for nearly 23.8% of the online social network market, against 56% of Facebook (May 2021). Even if it does not cover the majority of the population, Twitter has been shown in several contexts to be among the preferred channels for political communications [31].

We downloaded our data using Tweepy,^3^ a Python wrapper for the official Twitter API. The period that we considered goes from the 20th of November 2019 to the 23rd of December 2019 (the UK elections were held on the 12th of December). During this period, we collected all the tweets that contained the keyword *Brexit* using the GET /tweets/search/stream^4^ Twitter API, via Tweepy. We chose to limit the search to a single keyword since it is the specific term to indicate the departure of UK from EU and was ubiquitous in the political debate on this subject, such that as a neologism born in 2012, the word “Brexit” was added to Oxford dictionary in 2016.^5^

In order to overcome possible issues due to the usage of a neologism that appears unchanged in different languages, we filter the stream of tweets to English messages. We hit the 1% rate limit on data streaming only on 12th December, the election day. It could be argued that our filtering procedure is not adequate to analyse the whole discussion, as the UK general election also covered other topics of discussion that Brexit. However, our aim is to investigate the underlying sub-discussion on Brexit, as it played a central and polarising role, but also gained global attention from all over the world.

### Automated and suspended accounts

To assign a bot score to each account, we used the Botometer API [32] in its most recent v4 version [33]. Our choice is based on the recent comparison of results of various bot-detection algorithms, showing that Botometer has the best performances over recent annotated data sets [34]. Still, let us remark that Botometer can sometimes detect false positives/negatives and can have some general issues, see [35]. However, in our case we do not have many of the problems that are highlighted in the aforementioned paper, such as the language, which in our case is always English, and we are also using a more recent version of Botometer, which is performing better as also noted in [35]. For the sake of completeness, we provide the list of tweets (see Section Availability of data and materials).

Where not otherwise specified, all unverified users^6^ with a Botometer CAP (Complete Automation Probability) score greater or equal than 0.43 are considered bots, as was already chosen in [36]. There is no strict consensus on the threshold value that should be chosen. In our case, we chose the threshold so that the top 7 percentile of users will be labeled as bots. A similar approach was adopted by Ferrara et al. [37]. In Appendix A we show that the choice of the threshold does not modify the observed behaviour of the group, even if it changes the total number of automated accounts.

Furthermore, we decided to treat suspended users differently. In our analysis, we encountered a high number of accounts that were suspended (the Twitter API does not give more information). Dealing with these users, it is not possible to examine their past activity and give them a bot score, so we only had the posts that we downloaded. We decided to treat them as a separate class, because it is not granted that a user was suspended for violating the policies of Twitter on automation. Let us remark that the analysis of suspended users is also independent of the choice of bot detector. However, the analysis presented in [38] showed that the activity of suspended users presents many traits of malicious behaviours.

### Network models

*Network projection.* In order to analyse the bipartite networks we built with our data, we apply a recently-proposed algorithm to project the information contained in bipartite networks into monopartite ones [39]. The procedure consists in calculating, for each couple of nodes in one layer, the number of common neighbors on the opposite layer in the real systems, as a measure of similarity between nodes. Next, these observed similarities are compared to the expectations of an entropy-based null-model that accounts for the nodes’ degrees: if the observed similarity between the two nodes is statistically significant, a link connecting the two nodes is present in the one-mode projection.

We use the implementation proposed in [40] and the relative code of the package NEMtropy^7^ for the computation of the randomization and of the monopartite projection. In the following, the nodes corresponding to one layer will be called with the letter $r=1,\dots ,R$ as they correspond to the rows layer in a biadjacency matrix representation of the network, and the opposite layer will be indexed as $c=1,\dots ,C$ as they correspond to columns. We will call the biadjacency matrix representing the network with the letter **B** and its entries with $b_{rc}$.

More in detail, in a simple one-mode projection, we can produce a monopartite network of the nodes in one layer of a bipartite network by calculating the number of neighbors they share and setting that measurement as the weight of the link between them. The number of common neighbors is:

1 $$ V_{rr'}=\sum_{c}b_{rc}b_{r'c}= \sum_{c}V_{rr'}^{c}. $$

where we use $V_{rr'}^{c}\equiv b_{rc}b_{r'c}$ meaning that $V_{rr'}^{c}=1$ if and only if *c* shares a link to both *r* and $r'$. We then compare the measure of the similarity of the nodes to the similarity that is expected from a null-model that accounts for the nodes’ degrees. As a benchmark, we use the Bipartite Configuration Model (BiCM [41]), that belongs to the family of entropy-based null-models, [42–46]. These models arise from the maximisation of the Shannon entropy, which corresponds to maximising the uncertainty about the system, given some constraints, and resulting in a maximally unbiased model. Practically, the constrained maximisation of the Shannon entropy $S=-\sum_{\mathbf{B}}P(\mathbf{B})\ln P(\mathbf{B})$ leads to an exponential form for the probability distribution

2 $$ P(\mathbf{B})= \frac{e^{-H(\vec{\theta }, \vec{C}(\mathbf{B}))}}{Z(\vec{\theta })}, $$

where $\mathbf{C}(\mathbf{B})$ is the vector of constraints evaluated on the biadjacency matrix **B**, *θ⃗* is the vector of the Lagrangian multipliers associated to the maximisation procedure, $Z(\vec{\theta })$ is the partition function and $H(\vec{\theta }, \vec{C}(\mathbf{B}))$ is the Hamiltonian associated to the maximisation problem [42]. For each different case, the actual numerical probability $P(\mathbf{B})$ is determined by the targeted topological constraints that need to be accounted for [42]. In our case, we need to verify that the similarity between two nodes will be statistically significant when considering the respective degrees, so we employ the BiCM, that is the bipartite entropy-based null-model that takes as constraints the degree sequences of the two layers. With these constraints, the probability of a graph factorises in terms of probabilities per link:

3 $$ P(\mathbf{B})=\prod_{r=1}^{R}\prod _{c=1}^{C}p_{rc}^{m_{rc}}(1-p_{rc})^{1-m_{rc}}. $$

Each link probability will be a function of the $(R+C)$-vector of Lagrange multipliers *θ⃗* associated to the degrees as

4 $$ p_{rc}=\frac{x_{r}y_{c}}{1+x_{r}y_{c}}, $$

where $x_{r}=e^{-\theta _{r}}$ and $y_{c}=e^{-\theta _{c}}$ are reparameterizations of the Lagrange multipliers.

In order to find the numerical value of the probability, we set the average degrees of the model to the observed ones and solve the set of nonlinear equations

5 $$ \textstyle\begin{cases} \langle k_{r}\rangle =\sum_{c} p_{rc} = k_{r}^{*}, & r=1\dots R\\ \langle h_{c}\rangle =\sum_{r} p_{rc} = h_{c}^{*}, & c=1\dots C, \end{cases} $$

where $k_{r}$ and $h_{c}$ are the degree of the node *r* and *c* respectively, and ^∗^ indicates the observed empirical value. More details can be found in [40].

The entropy-based null-model allows to treat links as independent random variables, so the probability of a common neighbor reads $P(V_{rr'}^{c}=1)=p_{rc}p_{r'c}$. The distribution of a $V_{rr'}$ is then a sum of independent Bernoulli random variables with different coefficients, resulting in a Poisson-Binomial distribution. We then measure the statistical significance of the similarity of two nodes *r* and $r'$ by calculating the p-value of the observed $V_{rr'}^{*}$ with respect to the so obtained distribution. To perform this computation, given the large size of our datasets, we approximate the Poisson-Binomial distribution with a Poisson one with the same mean. The error of this approximation is controlled by Le Cam’s theorem [47–49]:

6 $$ \sum_{k=0}^{C} \biggl\vert f_{PB}(V_{rr'}=k)- \frac{\mu ^{k} \exp (-\mu )}{k!} \biggr\vert < 2\sum _{c=1}^{C}(p_{rc}p_{r'c})^{2}. $$

To validate links, we set a threshold that will distinguish the p-values that are significant from the ones to be disregarded. We apply the False Discovery Rate (FDR) procedure [50]. With $H_{1}, \dots H_{N}$ different hypotheses associated to different p-values, the recipe of FDR prescribes to sort the p-values in increasing order and then look for the largest integer *i* such that 7 where *t* is a single-test significance level (we use $t=0.05$ unless otherwise stated). Then we reject all hypotheses $H_{i}$ such that $i<\hat{i}$. In our case, the procedure validates all links with a p-value lower than the threshold found via the FDR.

**Figure 1:**
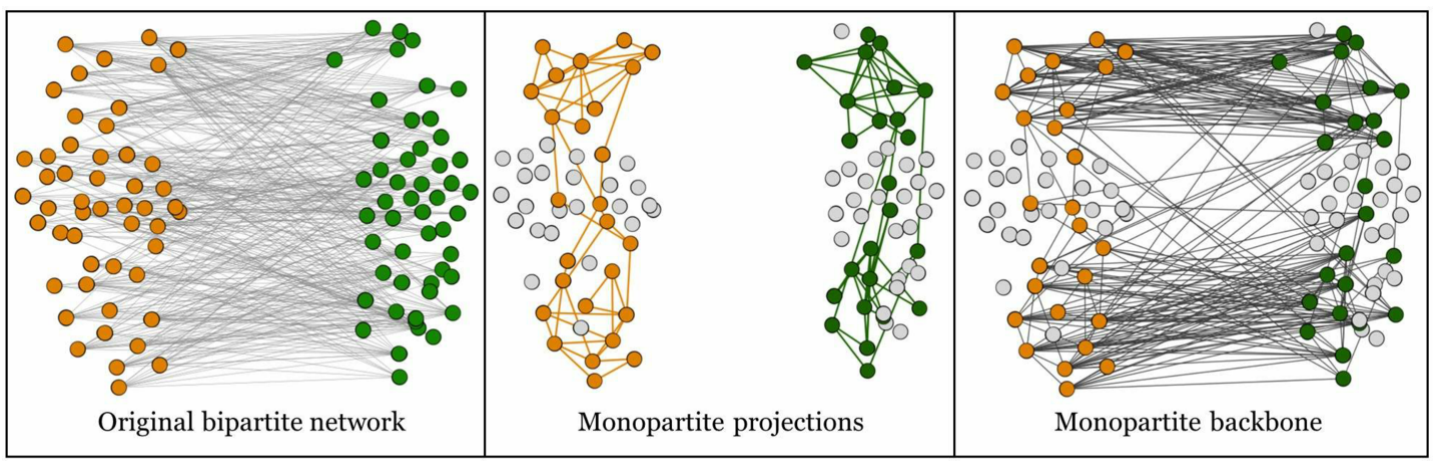
Monopartite backbone extraction from a bipartite network. The bipartite network of the first panel is projected on both layers. After the two one-mode projections are obtained (second panel), the original links can be added again to obtain the backbone of the network (third panel), that will highlight mixed groups of interactions

*Monopartite backbone.* After obtaining the two one-mode projections of a bipartite network, we add again the links of the original bipartite network to connect the nodes that remained in the projections. This way, we obtain a monopartite network that will show the groups of similar nodes of the same type, and link the groups if they were originally related. A representation of this procedure is shown in Fig. 1.

*Discursive communities.* A method for extracting memberships of users to different discursive communities in social networks was presented in [51] and later refined in [21, 52]. The method is based on the behaviour of verified users, i.e. the accounts that are certified by a Twitter official procedure: these are property of newspapers, newscasts, journalists, politicians, political parties and VIPs in general that can be of public interest. Verified users have a stronger tweeting (i.e. generation of original contents) activity than retweeting (i.e. sharing posts published by others) activity, on average. In this sense, we can leverage on the presence of verified users to extract discursive communities. In fact, we can use the validated projection described earlier and apply it to a bipartite network of verified and unverified users, in which a link is present if one of the users retweeted the other one at least once. In this sense, we are stating that two verified users are perceived as similar if they both interact with a considerable number of unverified accounts. After obtaining the validated projection, we can find different communities of verified users using the Louvain algorithm (see Sect. 2.4). With the so found community labels, we can obtain groups of similar unverified users in the original network by applying a label propagation algorithm. In this work, we simply label a user with the most common label among its neighbours, removing one edge at random in case of ties, using the algorithm proposed in [53] and considering the labels of verified users as fixed. Iterating the procedure several times until consensus is reached and no node will change its label, we obtain a labeling for all users. Since this procedure can yield different results for the random choice we make when breaking ties, we repeat the whole label propagation 1000 times and take the most frequent outcome. More details on this procedure can be found in [21, 51, 52].

### Community detection

To investigate the community structure of the networks, we use modularity-optimisation strategies. For monopartite networks, we look for optimal modularity partitions using the Louvain algorithm [54]. The Louvain community detection algorithm is order dependent [55], so we rerun the algorithm after reshuffling the order of the nodes. We then consider the partition in community displaying the largest value of the modularity.

### Core-periphery measures

To measure if nodes belong to the core or periphery of their communities, we employ a strategy similar to the one found in Guimerá et al. [56]. We use two measures: the first one, coming directly from this original paper, is called *participation score* and reads

8 $$ P(i) = 1 - \sum_{c=1}^{C} \biggl(\frac{k_{ic}}{k_{i}} \biggr)^{2} $$

where *i* is a node of the graph, *C* is the number of communities, $k_{i}$ is the degree of node *i* and $k_{ic}$ is the degree of node *i* towards the community *c*, i.e. the number of neighbors of *i* belonging to community *c*. The participation score will be 0 for a node that has all its neighbors in its own community, and $\frac{\overline{c} - 1}{\overline{c}}$ if its neighbors are all equally distributed inside *c̅* communities.

We also evaluate how relevant a node is inside its community, in accordance to the strategy of [56], but using a slightly different measure. In the original reference, in order to compare the degree of the nodes towards other nodes of their community, the authors propose the z-score of the in-degrees inside a community $c_{i}$ in the observed distribution. Actually, due to the non-Gaussian distribution of $k_{ic_{i}}$, the z-score is ill-defined and we prefer to introduce a new measure, that we call *relevance score*: 9

## Results

**Figure 2:**
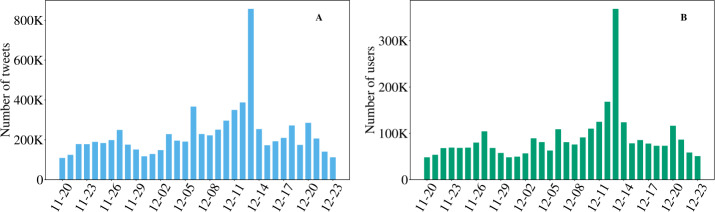
Number of tweets (including retweets) (**A**) and users (**B**) per day. The peak on the 12th of December that can be observed in both panels is in concurrence of the day of the elections

The number of posts per day fluctuates between 100K and 300K for most of the period we consider (Fig. 2), and it jumps to 900K on election day. In total, we analysed more than 10 million posts. The number of users follows a similar trend, being almost never lower than 50K and rising as high as 350K on the election day, for a total number of over one million users.

### Bots statistics

**Figure 3:**
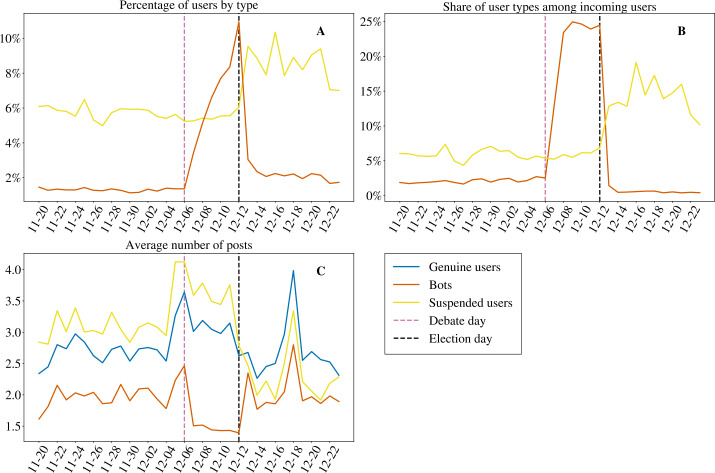
Presence of daily active bots and suspended users in the discussion over time. For users labeled as bots (with a score higher than 0.43) that have not been suspended, there is a change after the debate of the 6th of November (panel **A**), with new bots coming into the discussion (panel **B**). The new bots seem less active than the old ones (panel **C**). The percentage of active bots among genuine users becomes as high as 10%. Among the removed users, the changes happen after the 12th of December (election day), with new suspended users entering the discussion while being less active on average

In the following section, we quantify the number of bots in the dataset to attain RO1. The average percentage of bots in the discussion fluctuates around 2% for the first part of our time period (Fig. 3). Interestingly, though, the number of bots in the dataset increases steeply after the 6th of December. It rises as high as 11% on the election day, almost 10% higher than one week before, but rapidly drops after the election. The number of suspended users also shows a change of behaviour around that date, but a different one: their percentage goes up only moderately, from 6% to 9% after the election day, and this increase is not followed by a rapid drop. It can be observed that the increase in the bots’ presence comes with a relative decrease in their activity, as per Fig. 3, panel C: bots were, on average, tweeting more before the 6th of December, while the separation between users and bots increases from the 8th and they start to tweet less in the period of their increase before the elections. This behaviour comes mostly from retweet activity, so the bots that enter the Brexit discussion after the 6th of December seem to be more silent than the ones that were already present in the dataset.

For suspended users, the activity trends are almost inverted: they are more active than a normal user and considerably more than a bot, perhaps making it easier for Twitter to detect such accounts. Their activity does not show the same pattern of the bots but instead goes down after the election day.

As shown in Table 1, bots and suspended users tend to retweet malicious accounts more than genuine users, but they are not very supportive of each other and their activity focuses mainly on retweeting genuine accounts, thus generating noise and fostering discussions that are already present. Also, they are not much retweeted or quoted by genuine users, with only around 4% of the retweet/quote activity of genuine unverified users interacting with suspended users or bots, and even less for verified accounts. This suggests that the bots’ strategy is to mainly increase the visibility, and implicitly the credibility, of genuine accounts by retweeting their messages. The predilection of social bots for retweeting activities was also observed in US Covid-19 debate on Twitter [37]: the retweeting activity of social bots is more than 10 times their creation of new contents.

**Table 1:** Percentage of retweets and quote tweets by users, divided by type. Bots and suspended users are not very supportive of each other and their activity focuses on retweeting genuine accounts, thus generating noise and fostering discussions that are already present

User type	Tot %	Retweeted user type				Quoted user type			
		Suspended	Bots	Non-bots	Verified	Suspended	Bots	Non-bots	Verified
Suspended	8.58%	7.55%	1.18%	48.35%	42.92%	7.31%	0.82%	43.54%	48.33%
Bots	5.6%	3.86%	3.69%	43.85%	48.6%	2.18%	6.4%	34.17%	57.25%
Non-bots	84.51%	3.45%	0.72%	54.11%	41.72%	3.02%	0.56%	47.19%	49.23%
Verified	1.32%	0.82%	0.47%	34.43%	64.28%	1.25%	0.42%	26.06%	72.27%

The quantity of new bots that appear in the discussion can be seen in panel B of Fig. 3. The figure focuses on the new users and bots that appear for the first time in our dataset. To quantify the users that are appearing for the first time in the first days we have also used the tweets from the days before the 20th of November. The increase in the percentage of bots and suspended users comes with a higher relative percentage of new users of the corresponding type introduced in the discussion. This rapid, and large, increase of a new kind of bots in the dataset appears to be rather unique. With our analysis, we found differences between the Twitter activity of the already present bots and of the new bots. Remarkably, while the number of automated and suspended accounts increases (panel A of Fig. 3), their average activity does not present an analogous steep increase (panel C of Fig. 3): even if bots and suspended users enter overwhelmingly in the discussion, their activity remains nevertheless limited. Interestingly, the slopes of curves in panel C are quite similar, even if the levels are different. Otherwise stated, the different kinds of users seem to get a nearly constant share of total messages, while the total volume of posts changes. We also found significant differences in both panel A and B between the slopes of bots and suspended users, contributing towards RO4.

### Core-periphery structure and the bots’ position

To pursue RO2, we analysed the position of bots and suspended users in the networks of retweets. We obtained each network of retweets by linking two users if one of them has retweeted the other at least once, without considering the direction of the retweet nor their number. In this sense, the resulting network will be undirected and binary. By not considering the number of retweets, we avoid noise from accounts whose activity is much higher with respect to the others.

**Figure 4:**
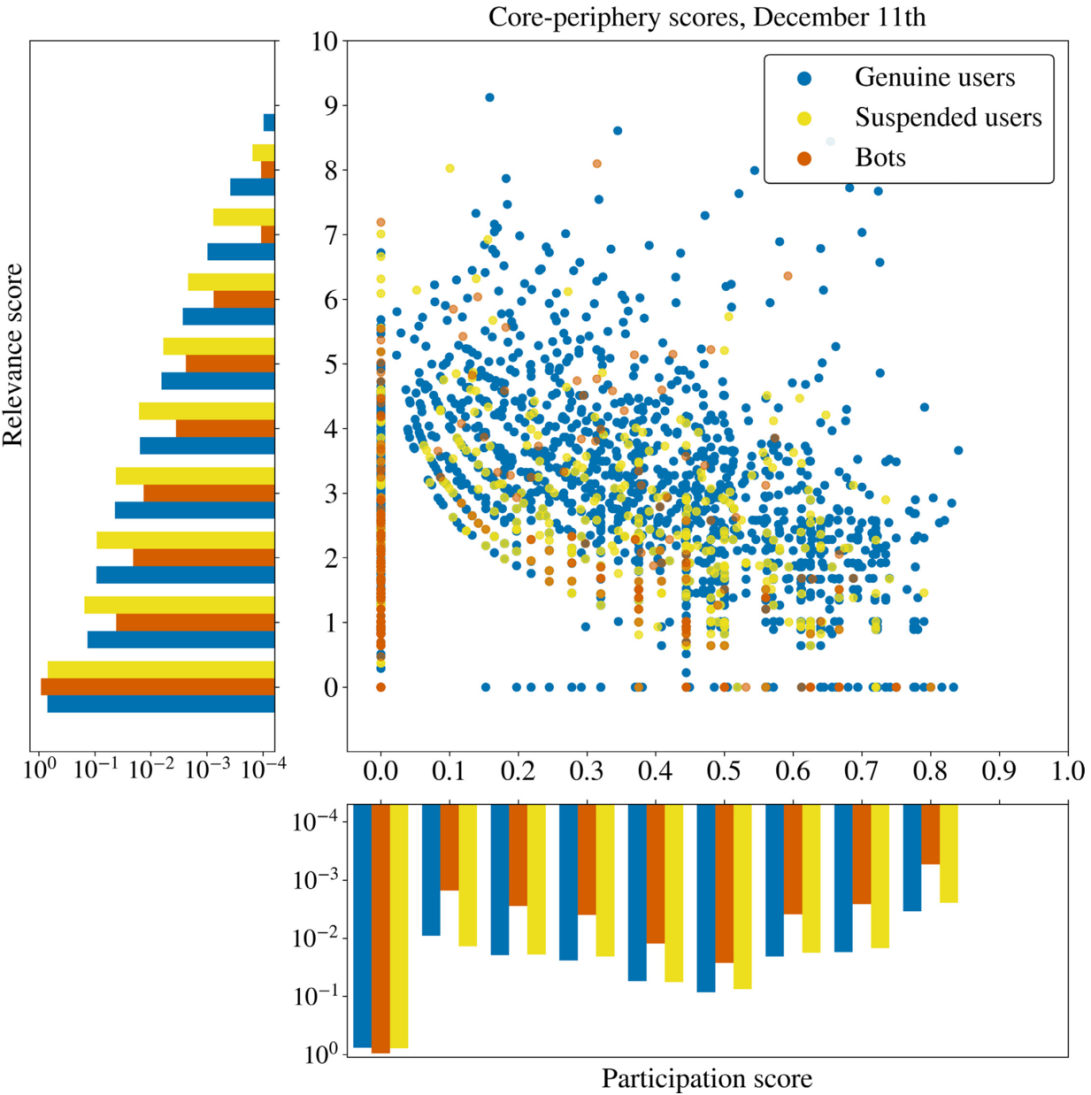
A scatter plot of the core-periphery scores with different colors for bots, suspended users and humans, for the network of retweets among users the day before the election. The histograms on the bottom and left of the scatter plot show the marginal distributions of the relevance score and participation score. While automated accounts are concentrated on lower values of presence and participation scores, suspended and genuine users have a flatter distribution, even if a peak on the lower values is still present. Bots are more inclined to retweet accounts in their community (low participation score) and moreover focus their activity on few users (low relevance score). The difference in core-periphery scores can be observed throughout the period we considered, see Appendix B for details

Our aim is to understand whether the retweet activity of the bots is strategically determined to fuel echo chambers or to target undecided users, by analysing the position of the bots in the network. We partially follow the approach used in [56] to measure the position of the nodes in the networks, and specifically whether the users lie in the core or periphery of their community. We employ a *participation score* (Eq. (8)) to measure how much a user restricts its retweet activity on only one community, and a *relevance score* (Eq. (9)) to measure how relevant a user is inside its community, see the Methods Sect. 2.5 for more details. The results are shown in Fig. 4: the bots, regardless of the community of belonging, seem to have a clear preference for focusing on retweeting users of their same community more than the other users. In the figure, we can see the distributions for the 11th of December. Indeed, the distributions of the *participation score* are very different when distinguishing between genuine users and bots, with a seemingly higher preference of bots for retweeting only in their own community, thus having a lower participation. The *relevance score* reveals instead a comparatively low centrality of the bots, meaning that the bots are usually on the periphery of one community, but focusing only on that single community with a low number of retweets, confirming the findings of [57]. The low scores of bots in both participation and relevance, however, can also be dependent on the degree distribution as well, since they very often tend to limit their activity to a single retweet or a few retweets. In Appendix B we show that the differences in the network position are consistent over time.

These results on core-periphery positions of the bots can also be explained by considering that the retweet activity of all users is not only focused on users of their same kind, as shown in Table 1. Indeed, bots and non-bots quote suspended users for a total of about 3-3.5% of their activity, while suspended users retweet and quote other suspended users for more than 7% of their total activity. A similar scenario appears for bots, whose retweet and quote activity focuses on bots much more (3.69% and 6.4%) than the activity of suspended and genuine users, who only direct towards bots around 1% of their total retweets. However, the automated accounts do not do enough to push a consistent part of them to the core of the retweet networks, leaving them more to the periphery than the other users.

### Discursive communities

To better understand the position of automated accounts and towards RO2, we analysed the bipartite network of verified and unverified users, which we obtained by linking two users if the unverified one has retweeted the verified user at least once. Understanding the structure of such network is useful to characterize the behaviour of accounts with respect to pivotal topics and their interactions with different political sides. With such a network, we analyse the discursive communities that were introduced in the Methods section.

**Figure 5:**
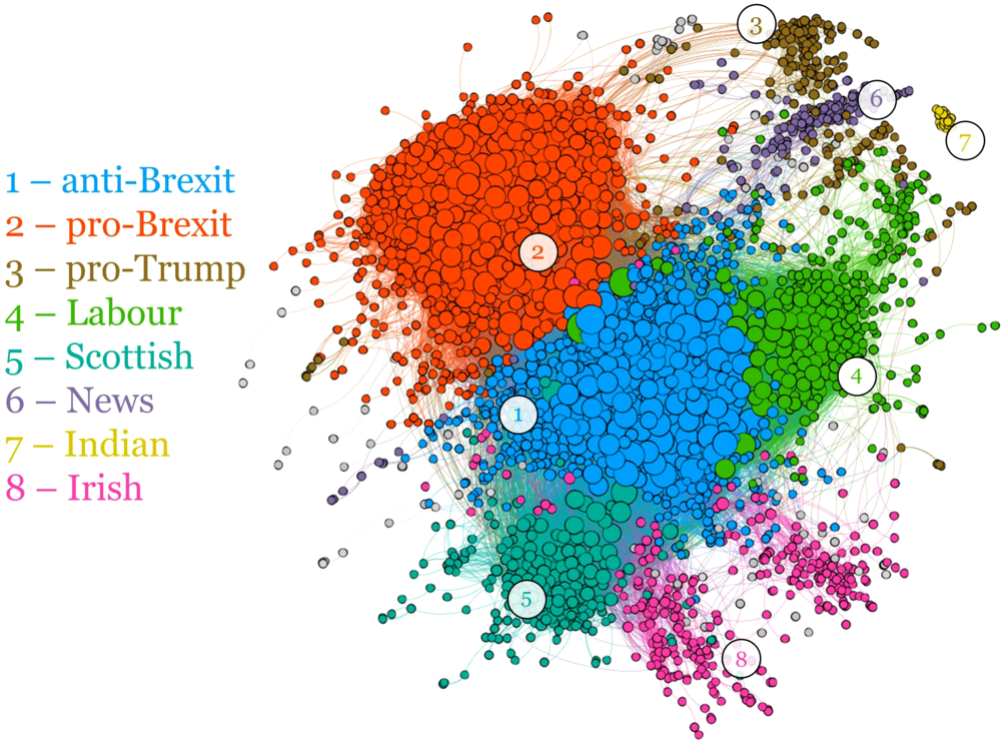
Projected network of the verified users. The eight biggest communities have been highlighted and their composition is further explained in Table 2

We first project the network on the layer of verified users by using the statistical projection described in Methods. We thus obtain a monopartite network of verified users (Fig. 5), that tells us which couples of verified accounts are retweeted by a significant number of common unverified users. With this network, we obtain labels for each verified user according to the community structure that we find by running the Louvain algorithm [54]. We gave a name to the communities *a posteriori* by looking directly at the users therein: indeed, the information about verified users is certified directly by Twitter platform and thus can be used to assess the nature of these communities. Then we are able to obtain the labels of unverified users by running a simple label propagation algorithm on the bipartite verified-unverified network. With the so found communities, we quantify the presence of bots and suspended users in each of them.

The results of this procedure can be read in Table 2. The communities have very straightforward interpretations, each containing verified users that can be associated with a stance on Brexit or with a country such as India or Ireland. Bots and suspended users are spread across the network and can be found in all communities, although their share varies consistently. The biggest community is the one supporting EU and against Brexit, with mixed users involved, including actors and famous people, but also politicians and parties such as *LibDems*. Then a pro-Brexit group includes the Conservatives and the Brexit Party. The pro-Brexit community has some interesting features. It presents a high percentage of suspended users, 11.76%, that also appear to be organized since their validation % is the highest among all groups. The validation percentage columns tell us how many users of a certain type are not discarded in the projection of the bots-hashtags and suspended users-hashtags networks (see Sect. 3.4), which in turn is a proxy for the structural coordination of the category of users. Moreover, the Brexit community has the highest link density (i.e. the ratio of the number of links over the number of users in the community) among all communities, meaning that the participation of users in this community is the highest. In general, the highest link densities are found in the groups that are directly involved with Brexit, i.e. pro- and anti-Brexit and the Scottish community; the Labour community link density is then surprisingly low. Remarkably, there is also a Trump supportive community, separated by the other groups and contains a staggering 23% of suspended users and a high coordination of the automated accounts shown by the 12% of validated bots, highest ratio among all groups, and the 7% of validated suspended users, second highest. The Labour party is remarkably forming a community by itself (i.e. separated from the no-Brexit one), and includes also some news accounts. It makes sense to find the Labour community far from the pro-Brexit side but on its own: it highlights the fact that the stance on Brexit of the Labour party was rather unique, since they were proposing a renegotiation of the Brexit deal and a new referendum. Many news accounts lie in a community of their own, linked to communities of both pro- and anti-Brexit stances, but it is not rare to find some news accounts inside other groups. Other smaller main communities are related to nationalities: Scottish, Indian and Irish in particular.

**Table 2:** Composition of the main discursive communities of the verified-unverified network after the label propagation. The projection on the verified users is shown in Fig. 5: from the projected network, the so-found communities are fixed labels in the verified-unverified bipartite network, and are then assigned to the rest of the users via label propagation. The *validated bots %* and *validated suspended %* columns show the percentages of bots and suspended users in the community that share a significant number of commonly retweeted hashtags with other users of the same type and therefore appear in the hashtags-users projections of Fig. 7: the higher these percentages are, the more the automated accounts are coordinated

#	Type	Size	Most retweeted verified users	% of bots	% of suspended users	% of verified users	% of validated bots	% of validated suspended users	Link density
1	anti-Brexit	479,718	davidschneider, DavidLammy, mrjamesob, Femi_Sorry, JimMFelton, HackedOffHugh, Channel4News, Keir_Starmer, LibDems	4.66%	4.71%	1.33%	3.16%	4.68%	7.9
2	pro-Brexit	166,254	BorisJohnson, Nigel_Farage, LeaveEUOfficial, Conservatives, brexitparty_uk, darrengrimes_, GoodwinMJ, KTHopkins, JuliaHB1	6.91%	11.76%	1.03%	2.91%	7.34%	12.72
3	pro-Trump	112,766	realDonaldTrump, ScottPresler, DiamondandSilk, RealCandaceO, ddale8, greggutfeld, DineshDSouza, IngrahamAngle, mtracey	3.7%	23.09%	0.71%	12.14%	7%	2.64
4	Labour	106,227	jeremycorbyn, OwenJones84, BBCPolitics, UKLabour, PeterStefanovi2, PeoplesMomentum, yanisvaroufakis, bbcquestiontime	8.79%	6.78%	0.75%	0.71%	1.92%	1.63
5	Scottish	20,258	theSNP, dannywallace, joannaccherry, NicolaSturgeon, IrvineWelsh, Feorlean, PeteWishart, AngusMacNeilSNP	5.35%	4.24%	1.6%	1.94%	5.47%	5.78
6	News	13,297	Reuters, BBCBreaking, business Brexit, TheEconomist, AJEnglish, AFP, nytimes, FT, CNN	8.92%	7.97%	4.93%	3.63%	1.13%	1.64
7	Indian	8908	gauravcsawant, swapan55, Iyervval, abhijitmajumder, AdityaRajKaul, TarekFatah, TVMohandasPai, WIONews, republic, samirsaran	1.95%	13.07%	0.67%	6.32%	4.3%	1.27
8	Irish	3323	SJAMcBride, naomi_long, sinnfeinireland, SenatorMarkDaly, GerryAdamsSF, ClaireHanna, Mr_JSheffield, cstross, glynmoody, rtenews	5.09%	3.49%	11.95%	3.55%	0%	1.41

Furthermore, Table 3 shows that the presence of automated accounts has also a different impact in different communities. In detail, the average total number of retweets per user across all our period is presented. When the number of retweets per user of bots and suspended users is high, the automated accounts are pushed more to the core of the discussion and can be more influential. Interestingly, in the pro-Trump community we find the bots with the highest number of retweets to their posts among all communities, with an average of almost 15 retweets to each bot. The second in terms of this measure is the pro-Brexit community that “only” has 5.7 retweets per bot. Instead, again the pro-Brexit community has the highest average number of retweets per suspended user.

**Table 3:** Average total number of retweets received by users of discursive communities of Fig. 5. In some communities, bots or suspended users are able to make themselves more credible and be retweeted much more: the bots of the pro-Trump community, for instance, are retweeted much more than in the other communities, while the suspended users of pro- and anti-Brexit communities get more attention than the others

#	Type	Average number of retweets received			
		Verified	Genuine	Bots	Suspended
1	anti-Brexit	1255.91	21.28	3.52	11.9
2	pro-Brexit	2924.08	23.14	5.71	19.53
3	pro-Trump	1777.63	4.34	14.59	3.5
4	Labour	3051.15	8.77	0.29	3.88
5	Scottish	1387.31	12.92	0.52	7.35
6	News	127.83	0.54	0.49	0.17
7	Indian	247.1	4.5	2.66	0.29
8	Irish	108.91	0.56	0.17	0.06

### What do the bots say?

Understanding the language used in online social networks is a crucial task with plenty of implications, especially in the case of automated accounts: in fact, a detailed characterization of the language used by social bots permits to study how automated accounts influence humans [19, 58]. Even if a detailed analysis of the language used by the different categories and its implications is beyond the scope of the present paper, we present some brief insights about the frequencies of hashtags used by the various user groups.

**Figure 6:**
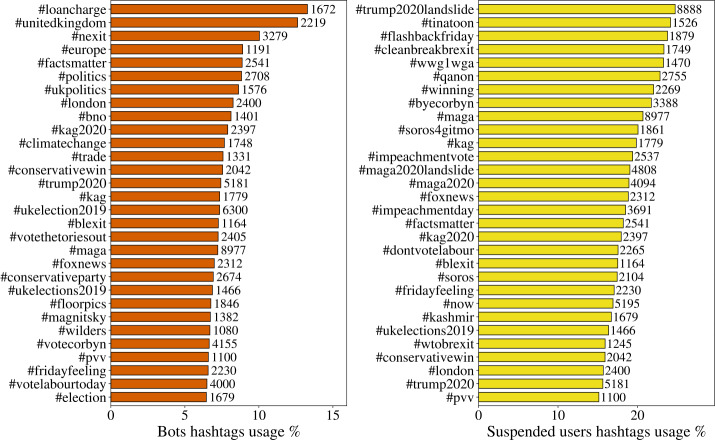
Most retweeted hashtags by bots and suspended users in percentage. The percentages are calculated over all users’ hashtags’ usage. The numbers on the right side of the bars represent the total number of retweets of posts (by all users) containing the corresponding hashtag

To understand what the bots are posting about for RO3, we observed the use of hashtags and URLs by users in our dataset. In Fig. 6 we show the most retweeted hashtags (in percentage) by bots and suspended users. On the right side of the bars, we also wrote the total number of posts found with each hashtag, to let the reader understand which hashtags, among those pushed by the bots/suspended users, are more central in the discussion. Interestingly, we can find topics that are external to Brexit, for example Trump 2020 campaign-related tags, suggesting an external connection of the Brexit debate to Trump’s campaign. Those hashtags are quite aggressive and target some US misinformation arguments (#Qanon, #soros4gitmo, #soros) or refer to the extreme right propaganda (#tinatoon is a popular comic strip of US alt-right; #blexit are black and latin-American Trump supporters that renamed themselves after the Brexit campaign). Nevertheless, suspended users’ hashtags referring to UK elections are supporting conservatives (#byecorbyn, #dontvotelabour, #conservativewin). Note that the use of popular hashtags such as #fridayfeeling in combination with political topics was observed also in [38].

In the activity of bots we still find several Trump-related hashtags (#kag2020, #trump2020, #blexit, #maga, #foxnews), even though we can see more focus on the actual theme of the UK election. In this case, the hashtags used by bots seem to be less prolarised: in fact, among the most used ones, we find, #votecorbyn, #conservativewin and #ukpolitics. #votethetoriesout and #conservativewin have nearly the same percentage. It is also worth to mention the presence of #nexit: the word Nexit is the equivalent of Brexit for the Netherlands.

**Figure 7:**
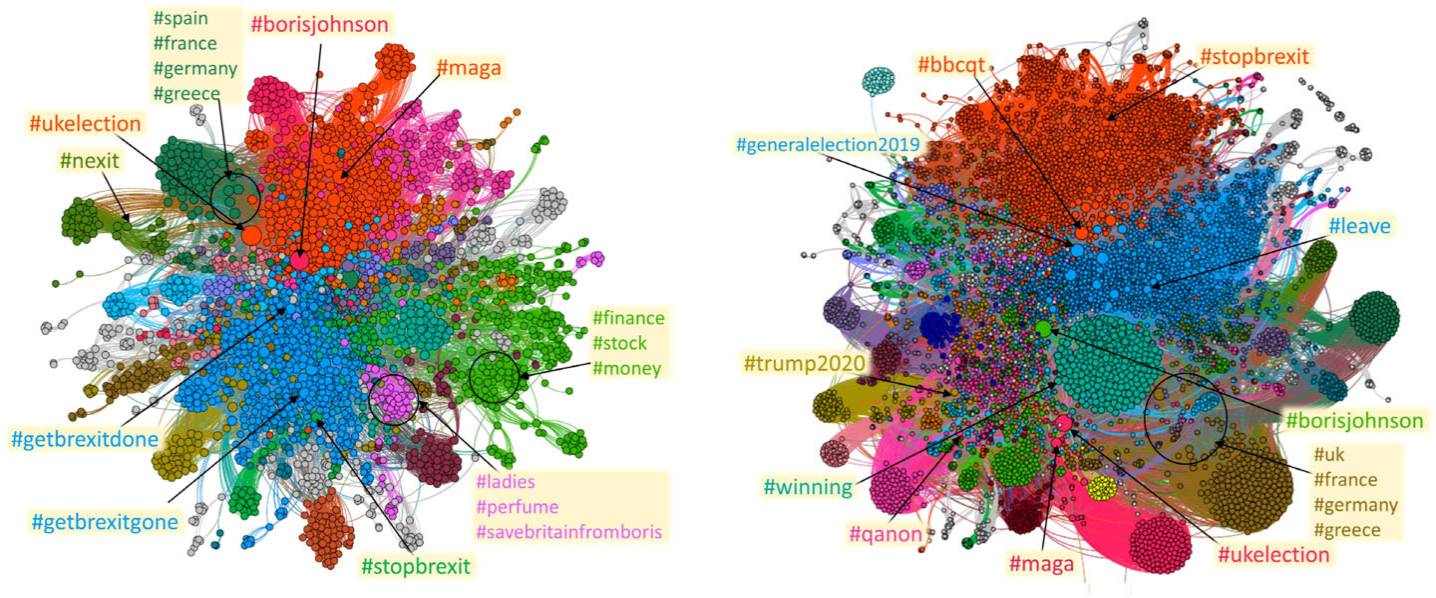
The backbones of the networks of hashtags and bots (left) and hashtags and suspended users (right) linked by retweets for the whole period of our dataset. Both networks show a modular structure probably due to the coordination of the automated users: the depicted partitions have a modularity of 0.73 (left) and 0.78 (right). In the bots’ network the Brexit discussion appears together in the blue community, while for the suspended users two separate groups are pro-Euro (orange) and pro-Brexit (blue). In both cases, Trump-related hashtags are very common

To improve our understanding of the bots’ organization, we built two bipartite networks connecting, respectively, bots and hashtags, and suspended users and hashtags. A connection is present between an account and a hashtag if the latter was retweeted at least once by the user. A first inspection suggests a separation of the activity of bots or suspended users in groups. In order to strengthen the signal, we extracted a backbone of the networks by projecting the bipartite network on its layers and re-linking the nodes of the two monopartite projections with the original links (see the Methods section for details). The two backbone networks can be seen in Fig. 7, with bots-hashtags on the left, and suspended-hashtags on the right. Our methodology reveals the dense cliques of hashtags that have common retweeters among bots and suspended users. We then search for communities in the network by running the Louvain algorithm for modularity optimization.

Among the mixed suspended users-hashtags communities highlighted in the right-side network of Fig. 7, there are some that are naturally linked together, and some that are most likely corresponding to an unusual activity of bots. For example, the orange and the blue communities, that are the largest ones, contain hashtags that refer to the elections and each one leans towards one direction (conservative/Brexit for the blue one, labour/pro-EU for the orange one). Meanwhile, some smaller but denser communities hashtags related to Qanon and the Trump campaign (pink, golden and purple, bottom left) or contain links to other EU countries such as France, Germany and Greece. For the network of bots, on the left of Fig. 7, the situation is similar but this time the discussion on Brexit is mixed in one community (blue), while the other communities contain either groups similar to those found for the suspended users, or other generic topics, maybe injecting noise or advertising products.

**Figure 8:**
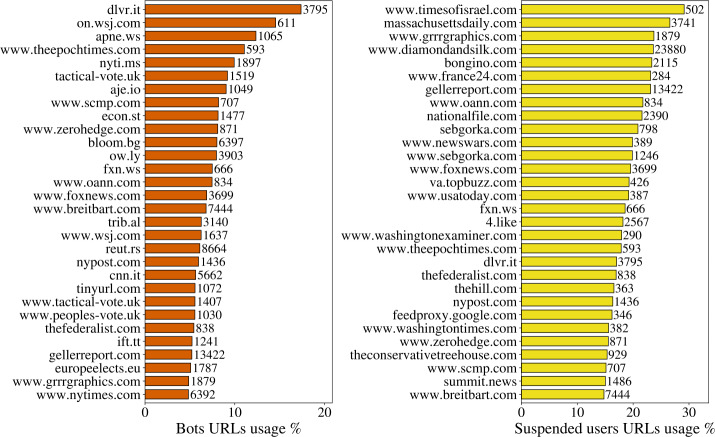
Most retweeted URLs by bots and suspended users in percentage. The percentages are calculated over all users’ URL’s usage. The numbers on the right side of the bars represent the total number of retweets of posts (by all users) containing the corresponding URL

Then we analysed the use of URLs by bots and suspended users. In Fig. 8 we present the URLs most used by bots and suspended users compared to genuine users. As in the case of hashtags, on the right side of each bar we also gave the total number of posts containing the corresponding URLs, to better show which of them are more central in the entire discussion. For example, *timesofisrael.com* appears in only a few hundreds retweets in total, most of them being retweets of a few tweets and is therefore not of particular interest in our analysis; conversely, *diamondandsilk.com* appears in more than 23K retweets. The retweet activity of both categories, but particularly suspended users, spams many websites that are close to Trump’s campaign, such as *diamondandsilk.com*, *gellerreport.com*, *breitbart*, *grrrgraphics*.

**Figure 9:**
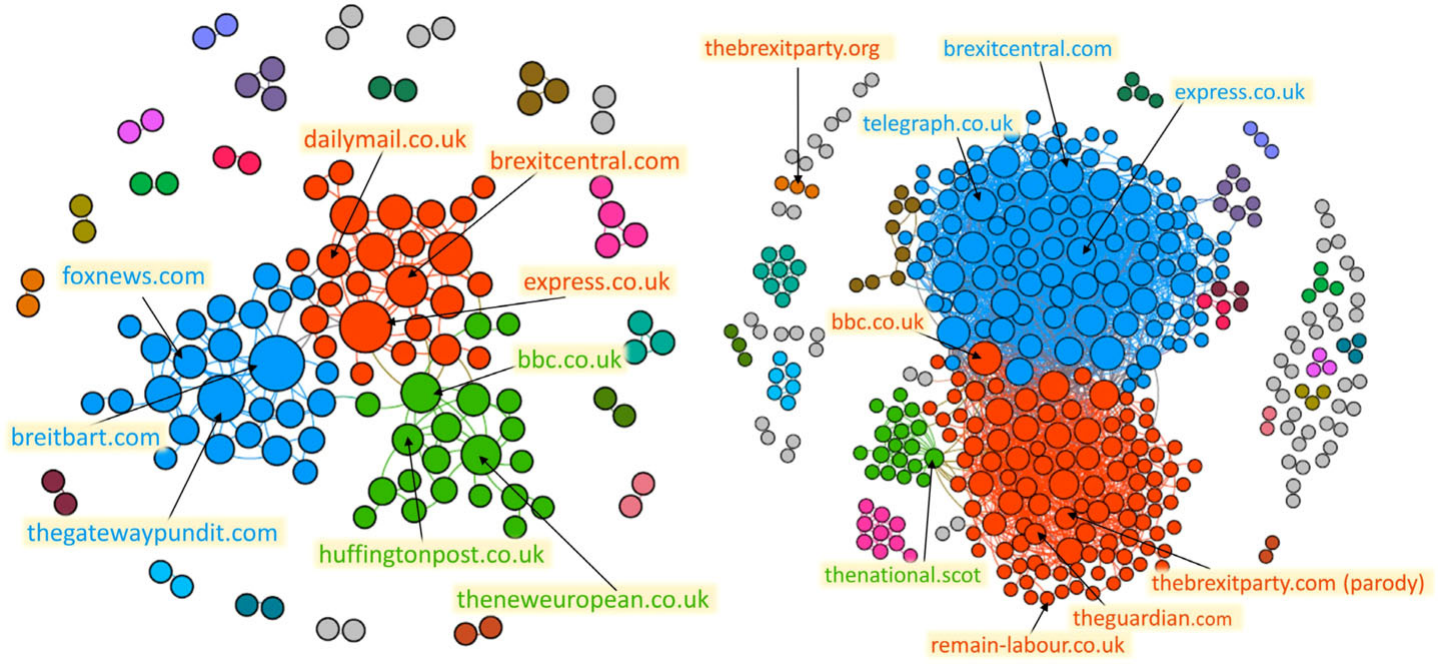
The projected networks of URLs used by bots (left) and by suspended users (right) for the whole period we consider. The suspended users are more organized in their patterns of retweets of URLs. Interestingly, Trump-related websites appear only in the bots’ projection

We also built the bots-URLs retweet network (Fig. 9) similarly to what we did for the hashtags, and in the same spirit we projected the network on the layer of the URLs via a statistical projection. Interestingly, on the projection from the URL-suspended users network we can find a separation of the URLs in the pro-EU and pro-Brexit links, and that is reflected also in newspapers URLs, with the most conservative ones leaning towards the blue community while the most pro-EU lie in the orange community. The green community on the right-side network in Fig. 9 is mostly made of Scottish websites. For the bots-URLs projection (left-side network of the same figure), although the projection is less populated, the story is similar, with a separation in pro-Euro (green) and pro-Brexit (blue) groups, but this time there is a community of Trump supportive websites of comparable size, including URLs and disinformation websites such as *diamondandsilk.com*, *gellerreport.com*, *breitbart.com* and such. This is analogous to what happened in the networks of bots/suspended users and hashtags. In this case, the projection on the users’ layer did not validate links between bots or suspended users since the use of URLs by users is way less diversified.

## Discussion and conclusion

Our analysis confirms that bots played a central role in the Brexit Twitter discussion.

We attained RO1 by quantifying the presence of bots in the discussion, in Sect. 3.1. We have observed that the percentage of bots went from less than 2% to more than 11% in one week, that is crucially the one just before the elections. This radical increase is observed right after the TV debate between the leaders of the main political parties, Boris Johnson and Jeremy Corbyn, which happened six days before the election. Such findings suggest that the injection of bots in the Brexit discussion was not a coincidence, but had the purpose of amplifying the importance of Brexit as a topic during the general election. Unfortunately, due to the limited time-window of our data, we cannot say if these bots are new to the whole political discussion or if they were simply shifting their attention on Brexit. Furthermore, we have observed differences in the temporal patterns of bots and suspended users. The presence of the suspended users is not increasing at the same time of bots, suggesting that one wave of bots went under the radar in Twitter’s bans, and that different types of automated accounts populate the Twitter landscape.

In Sect. 3.2, we contributed to RO2 by highlighting the differences in the activity of bots and genuine accounts and by analysing their different positions in the network structure. We started by considering the core-periphery structure of the network, partially using the approach of [56]: we analysed the position of the bots in the communities found in the networks of retweets, finding that automated accounts tend to be embedded in one single community and to stay in the periphery of it, confirming previous findings [19, 21, 57]. This is in part due to a large share of bots having low degree, but also to the fact that there were not many bots retweeting other bots, thus not being able to push some of the automated accounts to the core of communities. We also showed that the statistical differences between bots and humans increased with the mass insertion of bots. Furthermore, in Sect. 3.3, by considering the retweet patterns of unverified users towards verified users we were able to highlight groups of users tweeting about similar topics, and we analysed the participation of bots and suspended users in such groups. We found that the activity of bots is not limited to specific discussions, but instead they are present everywhere, although in different proportions. For suspended users instead, we found that a large part of their activity is focused on the Trump campaign. It is worth noting that Trump supportive bots have been observed even in French presidential election. Remarkably, in [9], the author detected that some automated accounts that supported 2016’s Trump presidential campaign and remained inactive in the following months, were later re-animated to support a disinformation campaign against Macron during French presidential campaign. Such a behaviour suggests, as noticed by the author, the possible existence of a black market for reusable political disinformation bots, also suggested in [59]. In the present manuscript we cannot state if the Trump-supporting bots and suspended users that we found have been used in other contexts, since we do not have any lists of bots active in other debates. Nonetheless, it is interesting to notice the presence this group displaying a so high number of particular efficient (due to their frequency of validated users) suspended accounts, again in a foreign political debate. Anyway, independently of their origin, we observed that bots and suspended accounts supporting Trump campaigns had a greater impact in the UK debate: in fact, Table 3 shows that the number of retweets per bots is much higher in the pro-Trump community than in any other one, even UK based. The different effects of US bots may be, as already suggested by the author of [9], due to the language continuity of UK, since it was absent in the case of French election campaign.

In Sect. 3.4 we dealt with RO3 by analysing the use of hashtags and URLs by bots. Our analysis has revealed that while there was a big activity by automated accounts, the streams of coordinated bots do not look to be only specifically linked to the Brexit discussion, but also on different topics that could benefit from the Brexit debate, mainly populist narratives. The two main topics that we uncovered are the Trump 2020 campaign, and perspectives of a further division in the EU.

Throughout our analysis, we treated the categories of bots and suspended users separately, in pursuit of RO4. In doing so, we were able to find very different patterns from the two types of agents. The topics most retweeted by both categories were quite similar, with a peculiar attention to populist topics and fake news websites. However, bots and suspended accounts are both injected in the conversation in mass, but not at the same time: bots increase radically in the days before the election and just after a pivotal debate, while the presence of suspended users grows rapidly after the election and is seemingly more linked to related populist topics or to Trump’s propaganda. Moreover, the levels of activity of the two categories were very different: our research shows that the accounts that have been suspended by Twitter were much more active than the automated accounts that were not suspended. This evidence could mean that the bots whose main activity consists in boosting one profile or topic through their retweets are able to go under cover much more than noisier accounts.

In achieving our ROs, we found distinguishable traits of the behaviour of bots. Accounting for false positives, as well as the limitation due to the choice of a threshold, are common concerns in bot detection; nevertheless, the users that we are labeling as bots present behaviours that are very close to those found in other studies [5, 8, 19, 36, 37, 57], thus we are reasonably confident about our results. Consider also that our analysis of suspended users is independent of the bot detection procedure and it contributes deeply to the narrative of our results.

The aim of our study was to characterize the presence of automated accounts on the “Brexit” subject during UK national election and identify their role in the debate. Summarising, we have found a significant presence of automated accounts in the Brexit discussion during the UK elections of 2019, following the findings of Bastos and Mercea [29] and Howard and Kollanyi [30] that also found a large presence of bots during the Brexit referendum. The activity of bots also presents a steep, apparently coordinated, increase, and this result is robust to the choice of the threshold used in the Botometer score. This increase comes at a crucial time, after a widely popular TV debate between political candidates one week before the elections and the elections themselves. We further analysed the behaviour of the automated accounts by analysing several network features, such as the core-periphery positions of users and the similarities of bots in the retweet patterns via the analysis of different kind of networks. We found that the automated accounts are spread across all the discussions. Furthermore, the bots behave significantly differently with respect to the genuine users, and we found groups of bots retweeting hashtags not always perfectly centred on the Brexit discussion but relative to other populist narratives. Remarkably, our findings corroborate some other results present in the literature about bot behaviour [5, 8, 19], for the specific context of UK election campaign. As already noted above, the presence of foreign social bots is significant: they were relatively easy to identify, since they used pro-Trump hashtags, together with pro-Brexit ones. Such an observation suggests the presence of an attention of bots’ masters to different events in various countries, as already observed in [9].

Due to the nature of our dataset, some questions remain unanswered. For instance, we do not know if the spike in bots’ activity is due to political bots shifting their attention on Brexit, as that would require a more general stream of tweets. However, further similar analyses on the specialisation of bots on a certain subtopic of a general discussion are needed to investigate these questions. Regarding methodological limits, while the general approach of entropy-based null-models is well grounded, the effectiveness of the extraction of discursive communities, even if it has been validated *a posteriori* with really good performances in different data sets [21, 51, 52, 60], has never been extensively tested. A comparison among different approaches is in preparation and suggests that the method implemented in the present manuscript is the one with the best performances [61].

## Figures and Tables

**Figure 10:**
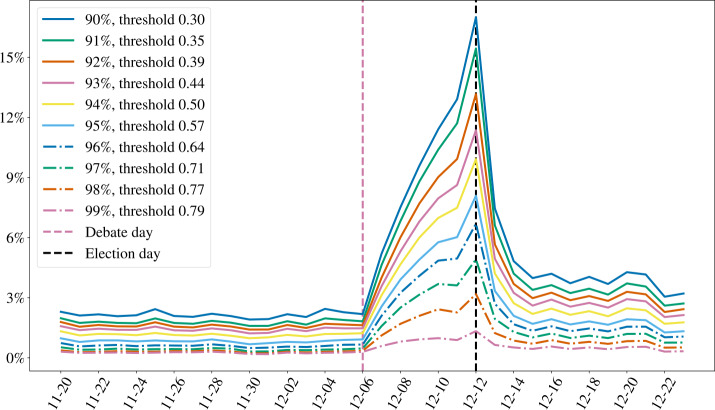
Percentage of bots per day obtained by setting different thresholds. Each threshold is chosen by considering as genuine a certain percentage of users over the entire period, as written on the legend. For instance, 90% of users are considered genuine (and the rest are bots) if the threshold is set at 0.3. The relative increase in bots’ presence appears independently of the chosen threshold

**Figure 11:**
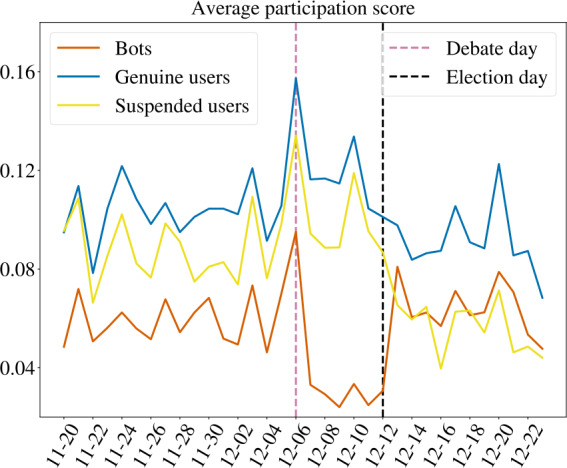
Average values of participation score by category. There is a sudden change of behaviour of bots after the election day in both the presence and participation score: in fact, the average values of the score are almost always higher for bots than for suspended users after the 12th of December. At the same time, the participation score of suspended accounts is always higher than average users before the election day, dropping to much lower levels after the elections

**Figure 12:**
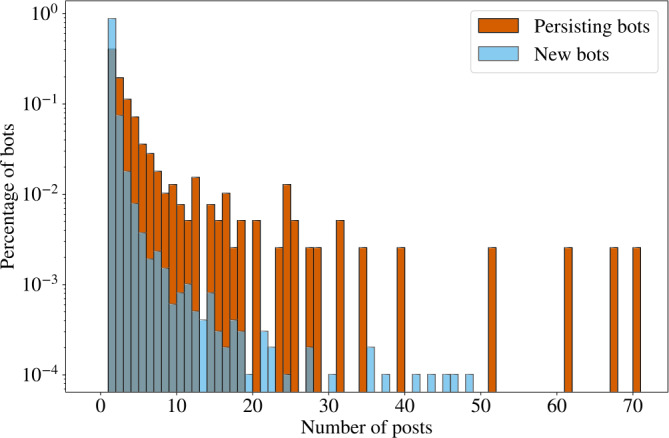
Histogram of the number of posts made by bots. We took bots from the 11th of December, and the two histograms separate the activity of those that were already present in the discussion the 6th of December (persisting bots) from the new entries (new bots). Almost 40% of those of the 6th of December are still in the discussion, and their average activity is almost stable from the two dates. The old users’ activity is much higher, so the effect of the less activity is only caused by the new bots’ behaviour

**Figure 13:**
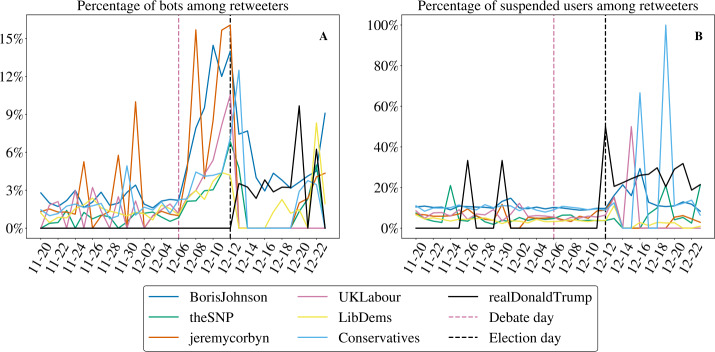
Percentage of bots (**A**) and suspended users (**B**) among retweeters for some main verified accounts. The percentages of suspended accounts increase after the election day, while in the case of bots the increase is observed after the day of the election

## Data Availability

The datasets used and/or analysed during the current study are available from the corresponding author on reasonable request. The list of all the tweets’ identifiers relative to this study is available at https://toffee.imtlucca.it/datasets.

## Appendix A: Choice of the CAP threshold in Botometer

It is remarkable that the pattern observed in Fig. 3 is independent of the threshold chosen to label users as bots: in Fig. 10 we show that the relative percentage increase of bots happens with different thresholds. We compare the number of bots detected each day by selecting different thresholds in order to have in total from 1% (threshold 0.79) to 10% (0.3) of users labeled as bots. In our case, we chose a threshold that was already used in [37] and labels as bots about 7% of all users in the dataset.

## Appendix B: Average participation scores

We computed the average participation score for the different categories of users over time (Fig. 11). The bots are almost always more focused on a single community than genuine users, and the difference increases in the week before the election. Suspended users behave similarly to the genuine users until the election day, after which they have scores more similar to the bots.

## Appendix C: Behaviour of incoming bots

It could be argued that the change of behaviour of the bots is not caused by the incoming automated accounts but rather by a change of behaviour of the ones that were already present in the dataset. Figure 12 suggests that this should not be the case. To explore this hypothesis, we separated the bots present on the 11th of December between the ones that were also present before the 6th of December, and the ones that were not. New bots are clearly less active, usually limiting their activity to a single retweet, while the ones that were already in the discussion show a more variegated activity.

## Appendix D: Automated retweeters of verified users

We also analysed what is the impact of bots and suspended users on the retweeters of some of the most popular verified accounts from various political parties. We found that the increase of bots is transversal across the users that we considered, while the suspended users’ increment seems to be driven by mainly *realDonaldTrump*’s retweeters, and partially *BorisJohnson*’s retweeters (Fig. 13).

## Footnotes

1 https://www.bbc.com/news/uk-politics-50303512

2 https://www.statista.com/statistics/280295/market-share-held-by-the-leading-social-networks-in-the-united-kingdom-uk/

3 https://www.tweepy.org/

4 https://developer.twitter.com/en/docs/twitter-api/tweets/filtered-stream/api-reference/get-tweets-search-stream

5 https://www.bbc.com/news/uk-england-oxfordshire-38326516

6 Twitter has a procedure to check the identity of some users that are of public interests, as the official account of political parties, newspaper, online newscasts, politicians, journalists and VIPs in general. Due to the verification procedure of Twitter, all verified accounts are considered as genuine by Botometer.

7 https://github.com/nicoloval/NEMtropy

## Abbreviations

OSN: Online Social Networks
RO: Research Objective
CAP: Complete Automation Probability
BiCM: Bipartite Configuration Model
FDR: False Discovery Rate
